# The Mediating Role of Endocrine Factors in the Positive Relationship Between Fat Mass and Bone Mineral Content in Children Aged 9–11 Years: The Physical Activity and Nutrition in Children Study

**DOI:** 10.3389/fendo.2022.850448

**Published:** 2022-03-24

**Authors:** Annie M. Constable, Dimitris Vlachopoulos, Alan R. Barker, Sarah A. Moore, Sonja Soininen, Eero A. Haapala, Juuso Väistö, Jarmo Jääskeläinen, Raimo Voutilainen, Seppo Auriola, Merja R. Häkkinen, Tomi Laitinen, Timo A. Lakka

**Affiliations:** ^1^ Children’s Health and Exercise Research Centre, University of Exeter, Exeter, United Kingdom; ^2^ Institute of Biomedicine, University of Eastern Finland, Kuopio, Finland; ^3^ School of Health and Human Performance, Dalhousie University, Halifax, NS, Canada; ^4^ Social and Health Center, City of Varkaus, Finland; ^5^ Faculty of Sport and Health Sciences, University of Jyväskylä, Jyväskylä, Finland; ^6^ Department of Paediatrics, University of Eastern Finland and Kuopio University Hospital, Kuopio, Finland; ^7^ School of Pharmacy, University of Eastern Finland, Kuopio, Finland; ^8^ Department of Clinical Physiology and Nuclear Medicine, Kuopio University Hospital, Kuopio, Finland; ^9^ Foundation for Research in Health Exercise and Nutrition, Kuopio Research Institute of Exercise Medicine, Kuopio, Finland

**Keywords:** adiposity, insulin, leptin, adiponectin, DXA (dual-energy X-ray absorptiometry), paediatric

## Abstract

**Introduction:**

We aimed to investigate whether the relationship between fat mass and bone mineral content (BMC) is mediated by insulin, leptin, adiponectin, dehydroepiandrosterone sulphate, testosterone and estradiol in children aged 9-11 years.

**Materials and Methods:**

We utilised cross-sectional data from the Physical Activity and Nutrition in Children study (n = 230 to 396; 112 to 203 girls). Fat mass and BMC were assessed with dual-energy X-ray absorptiometry. Endocrine factors were assessed from fasted blood samples. We applied the novel 4-way decomposition method to analyse associations between fat mass, endocrine factors, and BMC.

**Results:**

Fat mass was positively associated with BMC in girls (ß = 0.007 to 0.015, 95% confidence interval (CI) 0.005 to 0.020) and boys (ß = 0.009 to 0.015, 95% CI 0.005 to 0.019). The relationship between fat mass and BMC was mediated by free leptin index in girls (ß = -0.025, 95% CI -0.039 to -0.010) and boys (ß = -0.014, 95% CI -0.027 to -0.001). The relationship between fat mass and BMC was partially explained by mediated interaction between fat mass and free leptin index in boys (ß = -0.009, 95% CI -0.013 to -0.004) and by interaction between fat mass and adiponectin in girls (ß = -0.003, 95% CI -0.006 to -0.000).

**Conclusion:**

At greater levels of adiponectin and free leptin index, the fat mass and BMC relationship becomes less positive in girls and boys respectively. The positive association between fat mass with BMC was largely not explained by the endocrine factors we assessed.

**Clinical Trial Registration:**

[https://clinicaltrials.gov/ct2/show/NCT01803776], identifier NCT01803776.

## Introduction

Increased fat mass may increase bone mass through greater mechanical load on bones (1). As bone mineral content (BMC) tracks from pre-puberty through to adulthood, exploring the association between fat mass and BMC in pre- and early-pubertal children is important in understanding the factors that influence peak bone mass, and potentially lifelong skeletal health (1, 2). We have previously shown that in prepubertal girls and boys of largely normal weight status, fat mass was positively associated with areal bone mineral density (aBMD) independent of lean mass (3). Further, it has been shown that girls and boys with overweight and obese weight status had greater BMC than children with normal weight status after controlling for lean mass, in a predominantly pre- and early-pubertal sample (4). Longitudinally, fat mass at age 9 was positively associated with change in BMC over the following two years in pre-pubertal girls and boys independent of lean mass, though the weight status of the children was not characterised (5). The relationship between fat mass with bone mass is further supported by analysis with a Mendelian randomization approach in children aged 9 years, which found support for a positive causal relationship between fat mass and bone mass (6). Although evidence seems to indicate positive associations of fat mass with BMC and aBMD after controlling for lean mass in pre- and early-pubertal girls and boys with normal, overweight and obese weight status, the mechanisms underlying these associations are unclear. A review published in 2018 concluded that in early childhood fat mass may have a positive effect on developing bone, but in time this association attenuates and reverses so that especially excess fat mass with unfavourable metabolic changes may lead to detrimental effects on skeletal structure and strength (1).

In addition to the increased mechanical load on bones due to fat mass, adipose tissue is a regulator of endocrine function and secretes several hormones and cytokines that may influence bone metabolism both positively and negatively (1, 7). Insulin, leptin, dehydroepiandrosterone sulphate (DHEAS), testosterone and estradiol have been positively (8), and adiponectin negatively (3) associated with fat mass in largely normal weight pre-pubertal children. Insulin may increase bone mass by stimulating osteoblast differentiation (9). Leptin has a direct anabolic effect on bone, by driving the differentiation of bone marrow stem cells into osteoblasts, and an antiosteogenic indirect effect on bone, mediated *via* the sympathetic nervous system (1). Adiponectin may increase bone by driving osteoblast growth and inhibiting osteoclastogenesis, but also likely has negative indirect effects on bone, possibly by influencing the action of other hormones (10). Androgens and estrogens have a protective effect on bone by decreasing osteoclastogenesis, preventing osteoblast apoptosis and stimulating osteoclast apoptosis (11). Although the associations between these factors with bone have been investigated in clinical and adult populations, it is unclear whether these factors influence bone in healthy children (12–15).

Previous studies have hypothesized that the positive association between fat mass and bone mass in healthy pre- and early-pubertal children is mediated by endocrine factors, namely leptin and estradiol (4, 5). Of the studies which have considered endocrine factors, the positive association of fat mass with BMC was unchanged after controlling for markers of insulin resistance in female and male adolescents aged 15 years (16). This indicates that markers of insulin resistance do not mediate the association between fat mass and bone mass in adolescents. However, lean mass was not controlled for, and these findings cannot be extrapolated to younger children (16). In children and adolescents aged 8 to 14 years with overweight weight status and varying pubertal status, leptin was negatively associated with BMC, independently of weight, subcutaneous abdominal tissue, and intra-abdominal adipose tissue (17). However, lean mass was not controlled for, and these findings may not extend to children with healthy weight status, as leptin levels are positively associated with fat mass, and it has been suggested that the association between leptin with bone may be curvilinear (1). In girls and boys aged 9 years and females and males aged 15 years, adiponectin was negatively associated with BMC, controlling for fat mass and lean mass (18). This suggests an independent association of adiponectin with BMC in children and adolescents, though it is not clear whether adiponectin partially mediates the association between fat mass and BMC (18). As such, the mediating role of insulin, leptin, adiponectin, DHEAS, testosterone and estradiol in the relationship between fat mass with BMC in a population sample of healthy pre- and early-pubertal remains unclear.

Although the results of these studies suggest that endocrine factors may be important in the association between fat and bone, there are currently few and contradictory data on endocrine factors (4, 5, 16, 18). Studies to date have tended to use the ‘difference method’ for mediation analysis, which compares the association between the predictor and the outcome with and without taking the influence of potential mediators into account (16, 19). This method is limited in that it does not allow for interaction between exposures and mediators (19). Therefore, in cases where exposure-mediator interaction exists, but is not accounted for in the model, effect estimates are biased (19). These limitations can be addressed by applying the 4-way decomposition method, which decomposes the total association between an exposure with an outcome into a controlled direct association, a reference interaction, a mediated interaction, and a pure indirect association (20).

The aim of this study was to determine the association between fat mass and total body less head (TBLH) BMC in a population sample of girls and boys aged 9 to 11 years, and to assess the extent to which this relationship is mediated by insulin, leptin (free leptin index), adiponectin, DHEAS, testosterone and estradiol using the 4-way decomposition method. We hypothesized that fat mass would be positively associated with TBLH BMC, and that this association would be partially mediated by insulin, free leptin index, adiponectin, DHEAS, testosterone and estradiol.

## Materials and Methods

### Study Design and Participants

**Figure 1:**
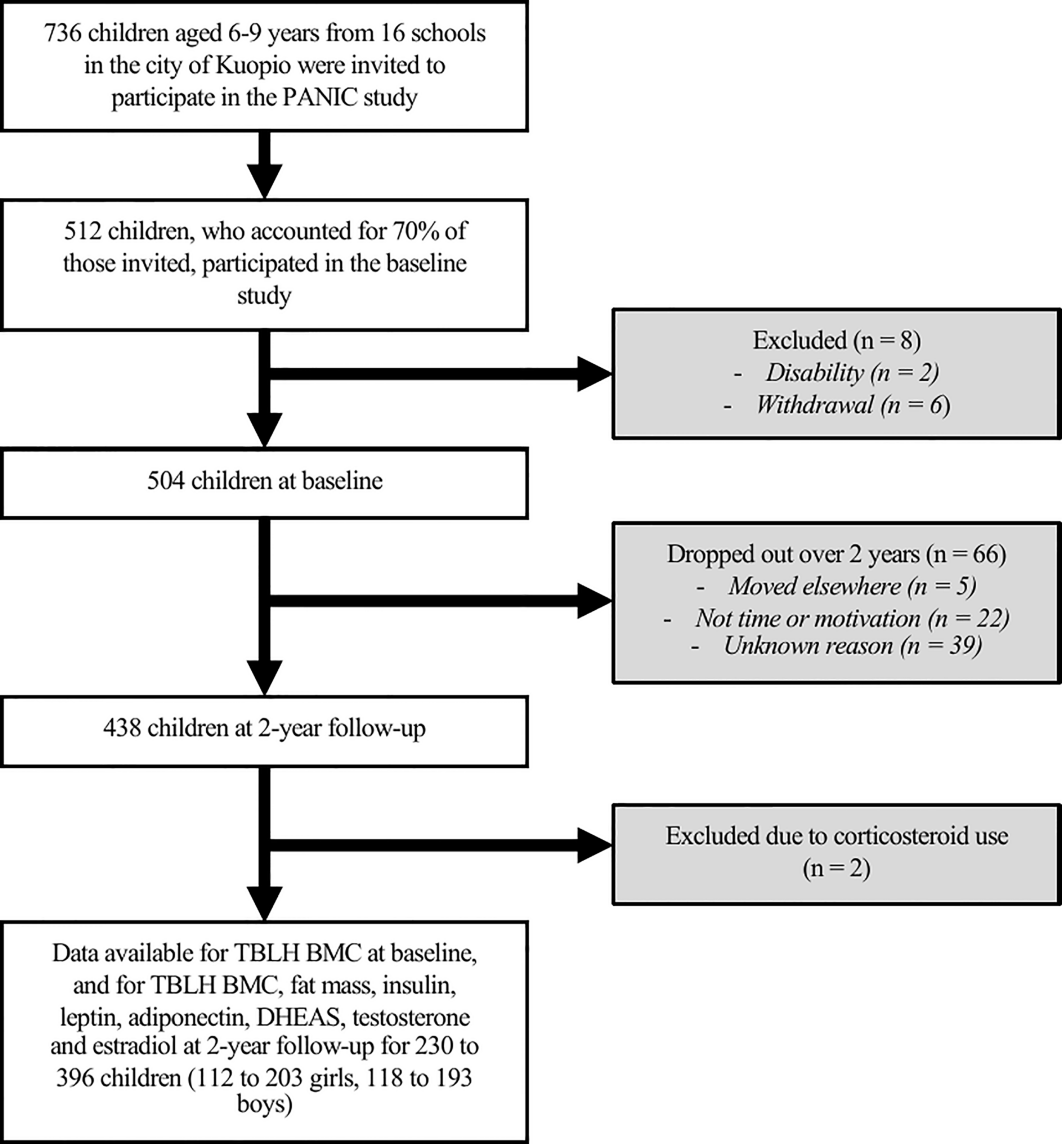
Participant flow chart. Physical Activity and Nutrition in Children, PANIC; Total body less head bone mineral content, TBLH BMC; Dehydroepiandrosterone sulphate, DHEAS.

This study utilized cross-sectional data from the two-year follow-up of the Physical Activity and Nutrition in Children (PANIC) Study, an 8-year controlled lifestyle intervention study in a population sample of Finnish children (ClinicalTrials.gov registration number NCT01803776) with ongoing follow-up assessments. BMC data were taken from baseline to include as a covariate. Of the 736 children invited to participate, 512 attended the baseline examinations and 438 attended 2-year follow-up examinations. For the present analyses, we excluded children who were currently taking or had previously taken oral corticosteroids, as this could influence BMC (21). Complete data on the main variables used in the present analyses were available for 230 to 396 children (112 to 203 girls, 118 to 193 boys). Inclusion and exclusion criteria are displayed in **Figure 1**.

The study protocol was approved by the Research Ethics Committee of the Hospital District of Northern Savo (approval number 69/2006). and conducted according to the ethical guidelines of the Declaration of Helsinki. The parents or caregivers of the children provided their written informed consent, and the children provided their assent to participation.

### Assessment of General Health and Pubertal Status

General health was assessed by a questionnaire completed by parents or caregivers. The questionnaire included items on children’s history of chronic diseases and allergies diagnosed by the child’s physician, and detailed information on children’s medications, prescribed to be used regularly or on demand. Pubertal status was assessed by a research physician. Central puberty was determined as breast development at Tanner stage ≥2 for girls and testicular volume ≥ 4 mL assessed by an orchidometer for boys (22).

### Anthropometry

Stature was measured in the Frankfurt plane with a wall-mounted stadiometer, three times to an accuracy of 0.1 cm. Body weight was measured twice using the InBody 720 bioelectrical impedance device (Biospace, Seoul, South Korea) to an accuracy of 0.1 kg. For stature and body weight, the mean of the values was used in analyses. Anthropometric measurements were conducted following the standard protocol used in Finnish health care, outlined by Saari et al. (23). Body mass index (BMI) (kg/m^2^) was calculated, and the BMI cut-offs of the International Obesity Task Force were applied to classify children as underweight, normal weight, overweight, or obese weight status (24).

### Assessment of Bone Mineral Content and Body Composition

TBLH BMC (g), aBMD (g/cm^2^), fat mass (kg) and lean mass (kg) were measured by a trained and experienced research nurse using the Lunar Prodigy Advance dual-energy x-ray absorptiometry (DXA) device (GE Medical Systems, Madison, WI) and the Encore software, Version 10.51.006 (GE Company, Madison, WI). These measurements were made in accordance with the instructions outlined by manufacturers using standardized protocols. The same DXA device and software were used for all measurements. DXA provides valid and reliable data on BMC and body composition in children (coefficient of variation = 0.01 – 4.37%) (25). A quality assurance test was performed daily prior to patient measurements. The inspection ensured the functionality and accuracy of the measuring device and method, tested the setting of the high voltage, the longitudinal and transverse movement of the imaging arc, the shutter mechanism of the beams and the accuracy of the detector. The inspection was performed using a recording table and a standard. A standard is a piece corresponding to a tissue material. Furthermore, for device repeatability monitoring a weekly testing was performed. According to the quality assurance program, the aluminum phantom was measured once a week. The acceptable measurement result in repeatability monitoring must not deviate by less than 2% from the long-term average. The effective dose in whole body scans was 1 to 3 mSv. The primary outcome variable was TBLH BMC, as evidence indicates that for pre- and early-pubertal children BMC is a more accurate and reliable measure than aBMD (26).

### Biochemical Analyses

Venous blood samples were taken following a 12-hour overnight fast. Blood was immediately centrifuged and stored at –75°C until biochemical analyses. Serum insulin concentration was measured by electrochemiluminescence immunoassay with the sandwich principle (Roche Diagnostics GmbH, Mannheim, Germany). Plasma leptin concentration was analysed using a competitive radioimmunoassay (Multigamma 1261-001, PerkinElmer Wallac Oy, Turku Finland) and plasma soluble leptin receptor concentration was analysed using an enzyme linked immunosorbent assay (ELISA) kit (Multicalc evaluation programme PerkinElmer Wallac Oy, Turku Finland). Free leptin index was calculated by dividing leptin with leptin receptor and multiplying by 100 and was used instead of leptin and leptin receptor in statistical analyses, as it has been suggested that free leptin index better reflects the physiological actions of leptin (27). Serum high-molecular-weight adiponectin concentration was analysed using an ELISA kit, following specific proteolytic digestion of other multimeric adiponectin forms (Millipore, Billerica, MA, USA). Serum DHEAS concentration was determined using an ELISA kit (Alpha Diagnostic International, San Antonio, TX, USA). Serum testosterone and estradiol were measured by using liquid chromatography-mass spectrometry (LC-MS), as explained in detail elsewhere (28). In the steroid analysis method, the lowest limit of quantitation of estradiol was 6.68 pmol/L, and therefore children with values below this were recorded as < 6.68 pmol/L. Although endocrine measures from a single fasted sample may not adequately reflect endocrine status across the day, a single fasted measure is common in epidemiological studies and in line with previous research (8, 16). The within-day and between-day coefficients of variation for the biochemical assays are provided in **Supplementary Table S2.1**.

### Statistical Analyses

Analyses were performed with Stata/SE for Mac software, Version 16.1 (StataCorp LLC, College Station, TX, USA). Differences in age, stature, BMI categories and pubertal status based on stages described by Tanner (22) between the included and excluded children were tested. Summary statistics were calculated for the total study sample, and for girls and boys separately. Independent samples t-tests, Mann-Whitney U tests and Chi-squared tests were used to test for sex differences.

**Figure 2:**
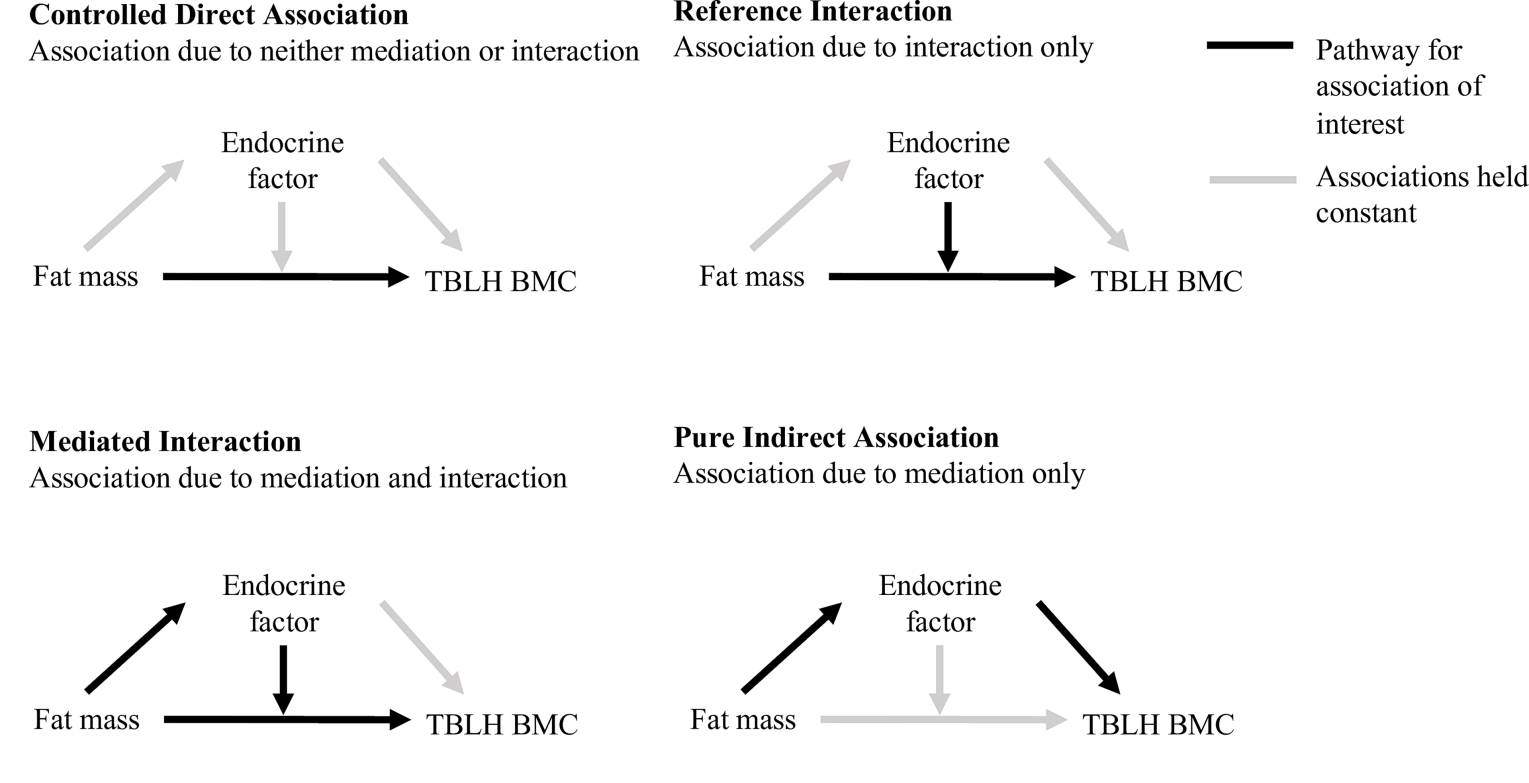
Components of the 4-way decomposition, as described by VanderWeele (20), of the total association between fat mass with TBLH BMC. Total body less head bone mineral content, TBLH BMC.

All variables were mean-centered prior to entry into regression models, and free leptin index was log-transformed to account for the curvilinear relationship with fat mass. We tested whether fat mass, insulin, free leptin index, adiponectin, DHEAS, testosterone and estradiol interacted with sex to influence TBLH BMC. As the insulin by sex by fat mass interaction and the adiponectin by sex by fat mass interaction were statistically significant, and as there was limited estradiol data for boys, further analyses were stratified by sex. We used the 4-way decomposition statistical approach with the *Med4Way* command in Stata/SE, which allows the total relationship between fat mass and BMC to be separated into four components: a controlled direct association (association due to neither mediation or interaction), a reference interaction (association explained by interaction only), a mediated interaction (association explained by mediation and interaction), and a pure indirect association (association due to mediation only) (see **Figure 2**) (20, 29). Two multivariable-adjusted regression models were established; an outcome model and a mediator model (see **Supplementary Material S1**). All models were adjusted for age, stature, pubertal status, lean mass, and baseline TBLH BMC (4, 30). The alpha value for statistical significance was set as 0.05. Effect estimates (ß), 95% confidence intervals (CI) and p-values were reported. It is common to report results of mediation analyses as the proportion of the association between the exposure and outcome which is mediated. However, in cases where the mediator-outcome association is in the opposite direction to the exposure-outcome association, to report the proportion mediated would be nonsensical, and is therefore not included.

Models were confirmed to meet assumptions of linearity, normality, and homoscedasticity of residuals. In 96% of boys, estradiol levels were under the lowest limit of quantitation, and therefore the analysis of estradiol could not be performed for boys. As 73% of girls had measurable levels of estradiol, the 27% of the samples with estradiol levels below the lowest limit of quantitation were included in analyses with the estradiol level coded as the lowest limit of quantitation. Given the nature of mediation analysis, multicollinearity is expected. In all models, all variance inflation factor (VIF) values were less than 10, and most were less than 5, indicating acceptable levels of multicollinearity (31). Influential outliers were assessed using Cook’s distance, and cases with Cook’s distance substantially greater than the rest were considered outliers (32). Outliers were observed in all models, so analyses were run with and without outliers to check whether the inclusion of outliers altered the results. For free leptin index, the inclusion of the eight children (3 girls, 5 boys) who were outliers altered the results. The alternative analysis strategy excluding these children is presented in **Supplementary Table S2.2**. For all other endocrine markers, the exclusion of outliers did not alter the results, so results are only presented including outliers.

## Results

### Characteristics of Children

Children included in this study did not differ from the children excluded from this study for age, stature, pubertal status and body weight status (**Supplementary Table S2.3**). For the children included in this study, stature, body weight, TBLH BMC and TBLH lean mass were lower in girls than boys, whereas TBLH fat mass, insulin, free leptin index, testosterone and estradiol were greater in girls than boys (**Table 1**). The proportion of pubertal children was greater in girls than boys.

**Table 1:** Descriptive characteristics of children.

	Total	Girls	Boys	P value for sex difference
	n = 396	n = 203	n = 193	
	Mean (SD)/Median (IQR)	Mean (SD)/Median (IQR)	Mean (SD)/Median (IQR)	
**Age (years)**	9.8 (0.4)	9.7 (0.4)	9.8 (0.4)	0.063
**Stature (cm)**	140.6 (6.3)	139.9 (6.5)	141.3 (6.0)	**0.021**
**Weight (kg)**	33.0 (29.4 to 38.9)	32.5 (28.7 to 37.4)	34.3 (29.9 to 34.5)	**0.028**
**BMI-SDS (kg/m^2^)**	-0.11 (1.06)	-0.13 (1.03)	-0.08 (1.09)	0.639
**Pubertal Status**				
**% (cases) prepubertal**	75.8 (300)	65.5 (133)	86.5 (167)	**<0.001**
**% (cases) pubertal**	24.2 (96)	34.5 (70)	13.5 (26)	
**IOTF Definition**				
**% (cases) thin and normal weight status**	82.3 (326)	83.7 (170)	80.8 (156)	0.447
**% (cases) overweight and obese weight status**	17.7 (70)	16.3 (33)	19.2 (37)	
**TBLH BMC (kg)**	0.92 (0.80 to 1.05)	0.88 (0.77 to 1.03)	0.94 (0.83 to 1.08)	**0.001**
**TBLH lean mass (kg)**	21.82 (2.96)	20.77 (2.78)	22.93 (2.75)	**<0.001**
**TBLH fat mass (kg)**	6.76 (4.33 to 11.13)	7.01(5.04 to 11.40)	6.48 (3.78 to 10.67)	**0.012**
**Insulin (mU/L)**	5.3 (3.8 to 7.8)	5.8 (4.1 to 8.3)	4.9 (3.3 to 7.1)	**0.001**
**Free leptin index**	15.3 (8.9 to 33.8)	17.6 (11.6 to 45.0)	12.6 (6.4 to 25.1)	**<0.001**
**Adiponectin (µg/ml)**	8.6 (4.1)	8.3 (3.7)	8.9 (4.6)	0.180
**DHEAS (µmol/L)**	0.7 (0.4 to 1.1)	0.7 (0.4 to 1.1)	0.7 (0.4 to 1.2)	0.536
**Testosterone (pmol/L)**	198.7 (137.5 to 291.9)	217.5 (154.3 to 323.9)	186.7 (118.4 to 264.6)	**0.014**
**Estradiol (pmol/L) *^a^***	<6.7 (<6.7 to 9.3)	9.4 (<6.7 to 19.4)	**<**6.7 (**<**6.7 to **<**6.7)	**<0.001**
**% (cases) with quantifiable levels (> 6.7 pmol/L)**	37.8 (87)	73.2 (82)	4.2 (5)	

Body mass index standard deviation score, BMI-SDS; International Obesity Task Force, IOTF; total body less head, TBLH; bone mineral content, BMC; Dehydroepiandrosterone sulphate, DHEAS.

a the quantitation limit of the assay is 6.7 pmol/L.

Number of children (n) varies from 230 to 396 for different variables;

n = 396, 203 girls and 193 boys: age, stature, weight, BMI-SDS, pubertal status, IOFT definition, TBLH BMC, TBLH lean mass, TBLH fat mass; n = 380, 194 girls and 186 boys: insulin; n = 377, 191 girls and 186 boys: adiponectin, n = 376, 190 girls and 186 boys: free leptin index, n = 374, 190 girls and 184 boys: DHEAS, n = 231, 113 girls and 118 boys: testosterone, n = 230, 112 girls and 118 boys: estradiol.

Bold emphasis indicates statistical significance with p < 0.05.

### Fat Mass

Fat mass was positively associated with TBLH BMC in girls (ß = 0.007 to 0.015, 95% CI = 0.005 to 0.020, *p* < 0.001) and boys (ß = 0.009 to 0.015, 95% CI = 0.005 to 0.019, *p* < 0.001) when adjusting for either insulin, free leptin index, adiponectin, DHEAS, testosterone or estradiol (**Supplementary Table S2.4**). When fat mass changed from the median to the 75^th^ percentile, the difference in TBLH BMC explained by the controlled direct association was positive in girls (ß = 0.034 to 0.068, 95% CI = 0.023 to 0.089, *p* < 0.001) and boys (ß = 0.034 to 0.064, 95% CI = 0.023 to 0.081, *p* < 0.001) in all models with either insulin, free leptin index, adiponectin, DHEAS, testosterone or estradiol (**Table 2**).

**Table 2:** Four-way decomposition of the association between fat mass and TBLH BMC.

	Girls			Boys		
	ß	95% CI	p-value	ß	95% CI	p-value
**Insulin**						
Total association	**0.035**	**0.025 to 0.046**	**<0.001**	**0.039**	**0.031 to 0.048**	**<0.001**
Controlled direct association	**0.034**	**0.023 to 0.044**	**<0.001**	**0.041**	**0.030 to 0.051**	**<0.001**
Reference interaction	0.000	-0.001 to 0.001	0.831	-0.000	-0.001 to 0.000	0.304
Mediated interaction	0.001	-0.000 to 0.003	0.085	-0.002	-0.005 to 0.000	0.101
Pure indirect association	0.001	-0.002 to 0.003	0.710	0.001	-0.004 to 0.007	0.630
**Free Leptin Index** ^a^						
Total association	**0.041**	**0.029 to 0.052**	**<0.001**	**0.042**	**0.034 to 0.050**	**<0.001**
Controlled direct association	**0.068**	**0.047 to 0.089**	**<0.001**	**0.064**	**0.047 to 0.081**	**<0.001**
Reference interaction	0.000	-0.000 to 0.001	0.407	0.000	-0.000 to 0.001	0.320
Mediated interaction	-0.003	-0.009 to 0.002	0.268	**-0.009**	**-0.013 to -0.004**	**<0.001**
Pure indirect association	**-0.025**	**-0.039 to -0.010**	**0.001**	**-0.014**	**-0.027 to -0.001**	**0.032**
**Adiponectin**						
Total association	**0.033**	**0.022 to 0.044**	**<0.001**	**0.038**	**0.029 to 0.046**	**<0.001**
Controlled direct association	**0.034**	**0.024 to 0.044**	**<0.001**	**0.037**	**0.029 to 0.045**	**<0.001**
Reference interaction	**-0.003**	**-0.006 to -0.000**	**0.031**	0.000	-0.002 to 0.003	0.759
Mediated interaction	0.002	-0.001 to 0.004	0.173	-0.000	-0.001 to 0.001	0.765
Pure indirect association	0.001	-0.001 to 0.002	0.434	0.001	-0.001 to 0.002	0.355
**DHEAS**						
Total association	**0.036**	**0.026 to 0.047**	**<0 001**	**0.037**	**0.028 to 0.045**	**<0.001**
Controlled direct association	**0.036**	**0.026 to 0.047**	**<0.001**	**0.036**	**0.027 to 0.045**	**<0.001**
Reference interaction	0.001	-0.001 to 0.002	0.268	0.001	-0.001 to 0.002	0.396
Mediated interaction	-0.001	-0.002 to 0.001	0.278	-0.000	-0.001 to 0.001	0.932
Pure indirect association	-0.000	-0.002 to 0.001	0.786	-0.000	-0.001 to 0.000	0.932
**Testosterone**						
Total association	**0.044**	**0.030 to 0.058**	**<0.001**	**0.035**	**0.024 to 0.045**	**<0.001**
Controlled direct association	**0.043**	**0.029 to 0.057**	**<0.001**	**0.034**	**0.023 to 0.044**	**<0.001**
Reference interaction	0.000	-0.001 to 0.002	0.828	0.000	-0.002 to 0.003	0.796
Mediated interaction	-0.000	-0.002 to 0.002	0.828	0.001	-0.003 to 0.004	0.785
Pure indirect association	0.001	-0.001 to 0.003	0.473	0.000	-0.002 to 0.003	0.760
**Estradiol**						
Total association	**0.040**	**0.026 to 0.054**	**<0.001**	.	.	.
Controlled direct association	**0.039**	**0.026 to 0.054**	**<0.001**	.	.	.
Reference interaction	0.003	-0.001 to 0.006	0.128	.	.	.
Mediated interaction	-0.002	-0.005 to 0.001	0.184	.	.	.
Pure indirect association	-0.001	-0.003 to 0.002	0.607	.	.	.

All models adjusted for age, stature, pubertal status, lean mass, and baseline TBLH BMC.

The values are effect estimates (ß), 95% confidence intervals (CI) and p-values from the 4-way decomposition model, as outline in **Figure 2**.

Total body less head bone mineral content, TBLH BMC; Dehydroepiandrosterone sulphate, DHEAS.

a Free Leptin Index log-transformed using the natural log.

The values at which the 4-way decomposition was computed are presented in **Supplementary Table S2.6**.

Number of children (n) varies from 230 to 380 for different variables:

Insulin: n = 380, 194 girls and 186 boys; Free Leptin Index, n = 376, 190 girls and 186 boys; Adiponectin; n = 377, 191 girls and 186 boys; DHEAS: n = 374, 190 girls and 184 boys; Testosterone; n = 231, 113 girls and 118 boys; Estradiol, n = 112, 112 girls and 0 boys.

Bold emphasis indicates statistical significance with p < 0.05.

### Insulin

Fat mass was positively associated with insulin in girls (ß = 0.260, 95% CI = 0.120 to 0.400, *p* < 0.001) and boys (ß = 0.406, 95% CI = 0.317 to 0.496, *p* < 0.001) (**Supplementary Table S2.5**). Insulin was not associated with TBLH BMC, and there was no interaction between fat mass and insulin with TBLH BMC (**Supplementary Table S2.4**). None of the total association between fat mass and TBLH BMC was mediated or moderated by insulin in girls or boys (**Table 2**).

### Free Leptin Index

Fat mass was positively associated with free leptin index in girls (ß = 0.186, 95% CI = 0.167 to 0.205, *p* < 0.001) and boys (ß = 0.177, 95% CI = 0.162 to 0.193, *p* < 0.001) (**Supplementary Table S2.5**). Free leptin index was negatively associated with TBLH BMC in girls (ß = -0.031, 95% CI = -0.049 to -0.014, *p* < 0.001) and boys (ß = -0.022, 95% CI = -0.039 to -0.004, *p* = 0.013) (**Supplementary Table S2.4**). There was an interaction between fat mass and free leptin index with TBLH BMC in boys only (ß = -0.003, 95% CI = -0.004 to -0.001, *p* < 0.001). When fat mass changed from the median to the 75^th^ percentile, the difference in TBLH BMC explained by the mediated interaction was negative in boys (ß = -0.009, 95% CI = -0.013 to -0.004, *p* = 0.031) (**Table 2**). The difference in TBLH BMC explained by the pure indirect association was negative in girls (ß = -0.025, 95% CI = -0.039 to -0.010, *p* = 0.001) and boys (ß = -0.014, 95% CI = -0.027 to -0.001, *p* = 0.032) (**Table 2**).

### Adiponectin

Fat mass was not associated with adiponectin in girls or boys (**Supplementary Table S2.5**). Adiponectin was negatively associated with TBLH BMC in girls only (ß = -0.002, 95% CI -0.004 to -0.000, *p* = 0.036), and there was an interaction between fat mass and adiponectin on TBLH BMC in girls only (ß = -0.001, 95% CI = -0.001 to -0.000, *p* < 0.001) (**Supplementary Table S2.4**). When fat mass changed from the median to the 75^th^ percentile, the difference in TBLH BMC explained by the reference interaction was negative in girls (ß = -0.003, 95% CI = -0.006 to -0.000, *p* = 0.031) (**Table 2**). None of the total association between fat mass and TBLH BMC was mediated or moderated in boys.

### DHEAS

Fat mass was not associated with DHEAS in girls or boys (**Supplementary Table S2.5**). DHEAS was not associated with TBLH BMC, and there was no interaction between fat mass and DHEAS with TBLH BMC in girls or boys (**Supplementary Table S2.4**). None of the total association between fat mass and TBLH BMC was mediated or moderated by DHEAS in girls or boys (**Table 2**).

### Testosterone

Fat mass was positively associated with testosterone in boys only (ß = 10.310, 95% CI = 0.089 to 20.531, *p* = 0.048) (**Supplementary Table S2.5**). Testosterone was not associated with TBLH BMC, and there was no interaction between fat mass and testosterone with TBLH BMC in girls or boys (**Supplementary Table S2.4**). None of the total association between fat mass and TBLH BMC was mediated or moderated by testosterone in girls or boys (**Table 2**).

### Estradiol

Fat mass was not associated with estradiol in girls (**Supplementary Table S2.5**). Estradiol was not associated with TBLH BMC, and there was no interaction between fat mass and estradiol with TBLH BMC in girls (**Supplementary Table S2.4**). None of the total association between fat mass and TBLH BMC was mediated or moderated by estradiol in girls (**Table 2**). Analysis in boys was not performed because they had values below the lowest limit of quantitation of estradiol (6.68 pmol/L).

**Figure 3:**
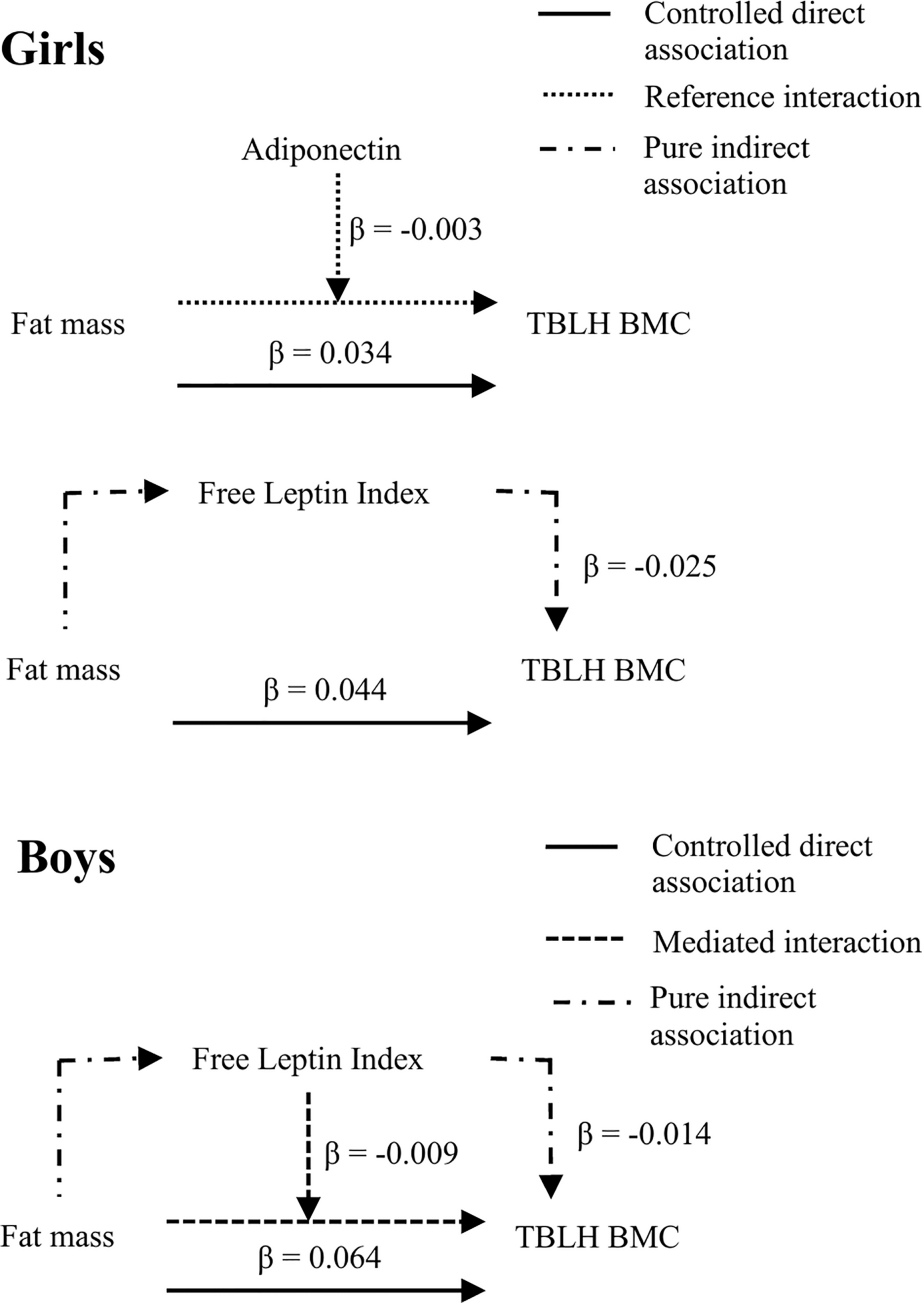
Summary of significant mediation and moderation effects from the 4-way decomposition. Total body less head bone mineral content, TBLH BMC. All models adjusted for age, stature, pubertal status, lean mass, and baseline TBLH BMC. Girls: n = 191 for adiponectin, n = 190 for free leptin index, Boys: n = 181.

A summary of the significant mediation and moderation effects is presented in **Figure 3**.

## Discussion

Our study is the first to formally test the mediation and interaction of insulin, free leptin index, adiponectin, DHEAS, testosterone and estradiol in the relationship between fat mass and TBLH BMC in a population sample of predominantly normal weight pre- and early-pubertal children. The positive relationship between fat mass and TBLH BMC was suppressed by mediation through free leptin index in girls and boys, and moderated by adiponectin and free leptin index in girls and boys respectively. Our findings indicate that the association between fat mass with TBLH BMC was largely not explained by the endocrine factors we assessed. Greater fat mass provides additional loading on bones, which may explain the positive association between fat mass with TBLH BMC. This proposed mechanism of mechanical loading due to increased fat mass is further supported by associations of a greater magnitude observed between lower-limb fat mass and BMC compared to upper-limb fat and BMC (unpublished observations), indicating the relationship between fat mass and BMC is more pronounced in load-bearing bones. However, as highlighted in a 2020 review article, the complex relationship between mechanical loading, due to lean mass, fat mass or weight-bearing activity, with bone metabolism, and the moderating role of associated endocrine and paracrine factors in this association is not yet fully understood (33).

The positive association between fat mass and TBLH BMC is in line with previous research in pre- and early-pubertal children (4, 5). The Avon Longitudinal Study of Parents and Children study demonstrated positive associations between fat mass and TBLH BMC in pre- and early-pubertal girls and boys aged 9.9 years, after accounting for lean mass (5). However, studies in adolescents and young adults have found negative (34) or null (35) associations between fat mass and BMC. This may reflect an interaction between fat mass and pubertal status, whereby the positive association between fat mass and TBLH BMC disappears or inverses during puberty (1). It may therefore be reasonable to suggest that pubertal status contributes to the differences observed between studies (4, 5, 34, 35).

### Insulin

*In vivo* evidence indicates that insulin acts as an anabolic agent for bone (12). However, similar to our findings, in children aged 7 to 15 years, Homeostatic Model Assessment for Insulin Resistance (HOMA-IR) did not meet the criteria for mediation with fat mass and TBLH BMC (36). Likewise, in females and males aged 15 to 16 years, although insulin was negatively associated with TBLH BMC, insulin did not mediate the fat mass and TBLH BMC relationship (16). The median levels of fasting insulin we observed were consistent with those observed in previous studies in the same age group (37), suggesting our sample was representative of children aged 9 to 11 years. Our observations replicate those of other studies, demonstrating that insulin does not mediate the association between fat mass and TBLH BMC (16, 36).

### Leptin and Free Leptin Index

Previous studies of the association between leptin and BMC have been inconclusive. *In vitro* studies indicate that leptin may stimulate peripherally-mediated osteogenic and hypothalamically-mediated antiosteogenic effects on bone (38). In children aged 7 to 8 years, plasma leptin was not associated with TBLH BMC (8). However, in children and adolescents aged 5 to 16 years, free leptin index was inversely associated with osteoprotegerin (38). Reduced osteoprotegerin may lead to increased bone resorption, potentially resulting in decreased bone mass. However, as bone mass was not measured in the aforementioned study, it is unclear whether the association between free leptin index and osteoprotegerin resulted in decreased bone mass (38). The pure indirect association we observed indicates that although the total association between fat mass with TBLH BMC was positive, the pathway through free leptin index acted as a suppressor in this relationship. The mediated interaction we observed indicates that free leptin index was positively associated with fat mass, and as fat mass increased, the relationship between fat mass with TBLH BMC became less positive in boys. It is therefore possible that in boys and male adolescents with greater levels of fat mass, the net association between fat mass with TBLH BMC may become negative (39). As around 80% of the participants in our study were a healthy weight, research in populations with a greater proportion of children and adolescents with overweight and obese weight status is needed to test this hypothesis.

The differences in the associations between fat mass, free leptin index and BMC between girls and boys may be due to the sex-specific patterns of leptin levels through childhood and adolescence, with greater absolute leptin concentrations observed in pre- and early-pubertal girls compared to boys (40, 41), which we also observed. However, as the free leptin index by sex by fat mass interaction was not statistically significant, the sex difference we observed may be superficial, due to reduced statistical power when stratifying the sample by sex. Differences in body composition, endocrine factors, as well as differences in pubertal status between girls and boys (40–42) may further explain the different associations in girls and boys between fat mass, free leptin index and TBLH BMC we observed.

### Adiponectin

Adiponectin has been inversely associated with fat mass in children aged 9.9 years (18). Further, adiponectin has been inversely associated with TBLH BMC at age 9.9 years and with the change in TBLH BMC from age 9.9 years to 15.5 years in the Avon Longitudinal Study of Parents and Children study cohort (18) independent of lean mass and fat mass. Similar to our findings, in adults adiponectin was inversely associated with BMC in females but not in males, potentially reflecting an influence of sex hormones on the action of adiponectin on bone (43). It is also possible that the sex difference we observed may be related to differences in fat mass between girls and boys, as girls had greater fat mass than boys in our sample. However, the reasons for the sex difference have not been formally tested, and it remains possible that our findings may be due to chance.

### DHEAS

Central adiposity has been positively associated with DHEAS, and children with obesity were more likely to have high DHEAS than normal weight children in a population sample of pre-pubertal children aged 7 years (44). Despite the positive association between obesity and premature adrenarche with advanced bone age (45), in children aged 7-8 years, DHEAS did not explain any additional variance in TBLH BMC beyond that of lean mass and fat mass (8). The median levels of DHEAS we observed were within the reference ranges for pre- and early-pubertal children (46), indicating our sample is representative of children aged 9 to 11 years. Therefore, our findings confirm previous evidence indicating that DHEAS does not have an independent association with bone when controlling for body composition.

### Testosterone and Estradiol

In children aged 7-8 years, fat mass was positively associated with testosterone and estradiol, though neither were associated with TBLH BMC, as was the case in our study (8). As three-quarters of our sample did not have signs of clinical puberty, it is possible that serum levels of sex steroids were too low for us to detect an effect. Although girls had higher levels of testosterone than boys, this is likely explained by the significantly greater proportion of pubertal girls compared to boys (47). We were also limited by the sensitivity of the estradiol measure, as 27% of girls and 96% of boys fell below the lower limit of quantitation in our sample. Previous studies have hypothesized that sex steroids may play a role in the positive associations between fat mass and bone mass in pre- and early-pubertal children (4, 5). Our findings do not support this hypothesis in pre- and early-pubertal children, though more sensitive measures of estradiol would be valuable in examining this further. The serum levels of testosterone and estradiol we observed are typical for pre- and early-pubertal children aged 9 to 11 years (46, 47). However, as sex steroid levels rise during puberty, further research into these relationships in adolescents is needed to understand the mediating role of sex steroids in the relationship between fat mass and BMC in adolescence.

### Relevance of Outcomes

We found across our outcome models that 1 kg greater fat mass was associated with around 0.01 kg greater TBLH BMC, after accounting for the effects of age, stature, pubertal status, lean mass, and baseline BMC. This increase seems to be modest, reflecting ~ 5 to 10% of the annual BMC accretion for children aged 9 to 11 years (48). The pure indirect association between free leptin index with TBLH BMC explained -0.025 kg difference in TBLH BMC in girls and -0.015 kg difference in TBLH BMC in boys, when fat mass increased from the median to the 75^th^ percentile. The mediated interaction between fat mass and free leptin index with TBLH BMC explained a -0.008 kg difference in TBLH BMC in boys only, when fat mass increased from the median to the 75^th^ percentile. The reference interaction between fat mass and adiponectin with TBLH BMC explained a -0.003 kg difference in girls, when fat mass increased from the median to the 75^th^ percentile, with adiponectin fixed at the median level. Although statistically significant, these associations represent a relatively small difference in absolute values. However, the negative interactions we observed may suggest that the observed estimates would be greater in a population with a greater proportion of children with overweight and obese weight status, though this requires further exploration.

### Strengths and Limitations

Strengths of this study included the population-based sample of children, the measurement of fat mass and bone outcomes by DXA, and the analysis strategy of controlling for baseline TBLH BMC at age 6 to 8. There are several limitations that should be considered when interpreting the results. The assessment of BMC is not simple, and the interpretation of results is challenging in growing children. The determinants of volumetric bone mineral density remain unclear, as it is not possible to obtain true measures for it from DXA. Therefore, the International Society for Clinical Densitometry recommends adjusting TBLH BMC using height z-score (49). We used TBLH BMC, as recommended by the International Society for Clinical Densitometry, and adjusted the data for age and stature, components of the height z-score, to account for body size (49). Future research with high-resolution peripheral quantitative computed tomography measures of bone micro-architecture would be valuable in further understanding the relationship between fat mass with bone in healthy children. The endocrine measures we used were from a fasted sample, which may not adequately reflect endocrine status across the day, due to diurnal changes in endocrine factors (50–52). This may lead to an under- or over-estimation of the association between endocrine factors and bone, though previous studies have also used fasted samples (8, 16). Further, although stature, age, pubertal status, lean mass, and baseline TBLH BMC were controlled for, residual confounding remains a potential limitation in our study and in all other observational studies.

Our findings indicate that fat mass was positively associated with BMC in pre- and early-pubertal children. Although adiponectin and free leptin index acted as mediators and moderators in the relationship between fat mass with BMC, these associations account for a relatively small contribution in terms of absolute values. These observations highlight that fat mass retains its positive relationship with BMC independently of the endocrine markers we assessed, and strategies to maintain bone mass, such as physical activity and nutrition, should be considered when recommending fat loss (53). As the relationship between fat mass and endocrine factors with BMC is likely moderated by weight status and pubertal stage (1), further research is needed to assess whether these observations extend to children and adolescents with overweight and obese weight status and with more advanced pubertal status.

## Data Availability Statement

The datasets presented in this article are not readily available because of research ethical reasons and because the owner of the data is the University of Eastern Finland and not the research group. Requests to access the datasets should be directed to www.panicstudy.fi/en/etusivu.

## Ethics Statement

The studies involving human participants were reviewed and approved by Research Ethics Committee of the Hospital District of Northern Savo. Written informed consent to participate in this study was provided by the participants’ legal guardian/next of kin and the participants provided their assent to participation.

## Author Contributions

AC, DV, AB, SM, SS, EH, and TAL contributed to the conception and design of the research. AC, DV, AB, SM, SS, EH, JV, JJ, RV, SA, MH, TL, and TAL contributed to the acquisition, analysis and interpretation of data. AC performed the statistical analysis and wrote the first draft of the manuscript. All authors contributed to manuscript revision, and all authors read and approved the submitted version.

## Funding

This work was financially supported by grants from Ministry of Social Affairs and Health of Finland, Ministry of Education and Culture of Finland, Finnish Innovation Fund Sitra, Social Insurance Institution of Finland, Finnish Cultural Foundation, Juho Vainio Foundation (application number: 202100397), Foundation for Paediatric Research, Doctoral Programs in Public Health, Paavo Nurmi Foundation, Paulo Foundation, Diabetes Research Foundation, The Finnish Medical Society Duodecim, Orion Research Foundation sr, Research Committee of the Kuopio University Hospital Catchment Area (State Research Funding), Kuopio University Hospital (previous state research funding (EVO), funding number 5031343) and the city of Kuopio. The UEF LC-MS Metabolomics laboratory is supported by Biocenter Finland and Biocenter Kuopio. The open access publication fees were supported by the University of Exeter.

## Conflict of Interest

The authors declare that the research was conducted in the absence of any commercial or financial relationships that could be construed as a potential conflict of interest.

## Publisher’s Note

All claims expressed in this article are solely those of the authors and do not necessarily represent those of their affiliated organizations, or those of the publisher, the editors and the reviewers. Any product that may be evaluated in this article, or claim that may be made by its manufacturer, is not guaranteed or endorsed by the publisher.
