# Supplementary material for: The Mediating Role of Endocrine Factors in the Positive Relationship Between Fat Mass and Bone Mineral Content in Children Aged 9–11 Years: The Physical Activity and Nutrition in Children Study
Source: Front Endocrinol (Lausanne). 2022 Mar 24;13:850448. doi: 10.3389/fendo.2022.850448 (PMC8987010; doi:10.3389/fendo.2022.850448)
Supplement: Supplementary file 1 [file DataSheet_1.pdf]

Supplementary Material

1 Supplementary Material

S1. Description of 4-way decomposition analysis

Two multivariable-adjusted regression models were established; an outcome model and a mediator model. Outcome models included fat mass, each endocrine factor, and an interaction term for fat mass and each endocrine factor as independent variables, and TBLH BMC as the dependent variable. Mediator models included fat mass as the independent variable and each endocrine factor as an outcome. As we used a continuous mediator, the mediator must be fixed at some level for the decomposition to be computed at (1). When the mediator is fixed at a given level, some of the effect of the interaction is captured by the controlled direct association. Therefore, the reference interaction should be interpreted as the proportion of the total association due to interaction between fat mass and each endocrine factor that is not captured by the controlled direct association and not mediated (1). The models and 4-way decomposition of effects are expressed as follows (2, 3):

Outcome Model: $E\,[Y \mid a, m, c] = \theta_0 + \theta_1 a + \theta_2 m + \theta_3 am + \theta_4' c$

Mediator Model: $E\,[M \mid a, c] = \beta_0 + \beta_1 a + \beta_2' c$

Where *a* is TBLH fat mass (i.e., exposure), M and *m* are the endocrine factors (i.e., mediators), *c* is a set of multiple potential confounders, and Y is TBLH BMC (i.e., outcome).

Based on the two regression models listed above, the controlled direct association, the pure indirect association, and the mediated interaction are as follows:

E [**Controlled Direct Association** $(m^*) \mid c] = (\theta_1 + \theta_3 m^*)(a - a^*)$

E [**Mediated Interaction** $\mid c] = \theta_3\beta_1 (a - a^*)\ (a - a^*)$

E [**Pure Indirect Association**$\mid c] = (\theta_2\beta_1 + \theta_3\beta_1 a^*)\ (a - a^*)$

Where m^* is a fixed mediator level at the median, a^* is the median level of TBLH fat mass, *a* is the 75th percentile of TBLH fat mass and c is a set of multiple potential confounders (age, stature, pubertal status, lean mass, baseline TBLH BMC).

The reference interaction is the difference between the pure direct association, given by E [**Pure Direct Association** $\mid c] = \{\ \theta_1 + \theta_3(\beta_0 + \beta_1 a^* + \beta_2' c)\}\ (a - a^*)$, and the controlled direct association:

E [**Reference Interaction** $(m^*) \mid c] = \theta_3\ (\beta_0 + \beta_1 a^* + \beta_2' c - m^*)\ (a - a^*)$

Estimates for the 4-way decomposition were computed fixing fat mass at the median and 75th percentile and endocrine factors at medians (Supplementary Table S2.6).

2 Supplementary Tables

Table S2.1. Within-day and between-day coefficients of variation for measurement of endocrine factors

	**Within-day coefficient of variation**	**Between-day coefficient of variation**
Serum insulin	1% to 4%	2% to 4%
Plasma leptin	2%	4%
Plasma soluble leptin receptor	7%	5%
High-molecular-weight adiponectin	2%	17%
DHEAS	9%	12%
Testosterone	4%	6%
Estradiol	6%	9%

Dehydroepiandrosterone sulphate, DHEAS.

Table S2.2 Alternative analysis strategy for 4-way decomposition of the association between fat mass and TBLH BMC excluding outliers

	**Girls**			**Boys**		
	ß	95% CI	p-value	ß	95% CI	p-value
**Free Leptin Index**[a]						
TE	**0.036**	**0.026 to 0.046**	**< 0.001**	**0.033**	**0.025 to 0.040**	**< 0.001**
CDE	**0.052**	**0.032 to 0.072**	**< 0.001**	**0.044**	**0.027 to 0.061**	**< 0.001**
INTref	0.000	-0.000 to 0.001	0.530	0.001	-0.000 to 0.001	0.240
INTmed	-0.003	-0.010 to 0.004	0.335	**-0.007**	**-0.013 to -0.002**	**0.007**
PIE	-0.013	-0.027 to 0.001	0.063	-0.004	-0.018 to 0.010	0.539

All models adjusted for age, stature, pubertal status, lean mass, and baseline TBLH BMC.

Total body less head bone mineral content, TBLH BMC; total effect, TE; controlled direct effect, CDE; reference interaction, INTref; mediated interaction, INTmed; pure indirect effect, PIE.

[a] Free Leptin Index log-transformed using the natural log.

The values at which the 4-way decomposition was computed are presented in Supplementary Table S2.2.

n = 368, 187 girls and 181 boys.

Table S2.3. Differences in participant characteristics between included and excluded children

	Included children	Excluded children	P value for group difference
	n = 396	n = 108	
	Mean (SD) / Median (IQR)	Mean (SD) / Median (IQR)	
**Age (years)**	9.76 (0.43)	9.68 (0.45)	0.246
**Stature (cm)**	140.60 (6.29)	139.15 (6.27)	0.161
**Weight (kg)**	9.76 (9.45 to 10.02)	9.60 (9.39 to 9.98)	0.111
**BMI-SDS**	-0.05 (-0.93 to 0.62)	-0.52 (-1.02 to 0.34)	0.166
**Pubertal Status**			
**% (cases) prepubertal**	75.8 (300)	91.7 (22)	0.074
**% (cases) pubertal**	24.2 (96)	8.3 (2)	
**IOTF Definition**			
**% (cases) normal weight**	82.8 (328)	82.9 (34)	0.414
**% (cases) overweight**	13.6 (54)	17.1 (7)	
**% (cases) obese**	3.6 (14)	0 (0)	

Body mass index standard deviation score, BMI-SDS; International Obesity Task Force, IOTF.

Number of children (n) varies for different variables.

n = 437, 396 included and 41 excluded: age, stature, weight, BMI-SDS, IOFT definition; n = 420, 396 included and 24 excluded: pubertal status.

Table S2.4. Associations between fat mass, endocrine factors and total body less head bone mineral content (outcome model)

Endocrine Factors	Variables included in the Model	Girls		Boys	
		ß (95% CI)	p-value	ß (95% CI)	p-value
**Insulin**	Fat mass	**0.008 (0.006 to 0.010)**	**< 0.001**	**0.009 (0.007 to 0.012)**	**< 0.001**
	Insulin	0.001 (-0.002 to 0.003)	0.479	0.000 (-0.003 to 0.003)	0.816
	Fat mass X Insulin	0.000 (0.000 to 0.001)	0.050	-0.000 (-0.001 to 0.000)	0.096
**Free Leptin Index[a]**	Fat mass	**0.015 (0.012 to 0.020)**	**< 0.001**	**0.015 (0.011 to 0.019)**	**< 0.001**
	Free Leptin Index	**-0.031 (-0.049 to -0.014)**	**< 0.001**	**-0.022 (-0.039 to -0.004)**	**0.013**
	Fat mass X Free Leptin Index	-0.001 (-0.002 to 0.001)	0.267	**-0.003 (-0.004 to -0.001)**	**< 0.001**
**Adiponectin**	Fat mass	**0.007 (0.005 to 0.010)**	**< 0.001**	**0.009 (0.007 to 0.011)**	**< 0.001**
	Adiponectin	**-0.002 (-0.004 to -0.000)**	**0.036**	-0.001 (-0.003 to 0.001)	0.179
	Fat mass X Adiponectin	**-0.001 (-0.001 to -0.000)**	**< 0.001**	0.000 (-0.000 to 0.000)	0.758
**DHEAS**	Fat mass	**0.009 (0.006 to 0.011)**	**< 0.001**	**0.008 (0.007 to 0.010)**	**< 0.001**
	DHEAS	0.005 (-0.008 to 0.017)	0.481	0.006 (-0.005 to 0.016)	0.275
	Fat mass X DHEAS	0.002 (-0.001 to 0.004)	0.142	0.001 (-0.001 to 0.003)	0.217
**Testosterone**	Fat mass	**0.009 (0.007 to 0.013)**	**<0.001**	**0.008 (0.005 to 0.010)**	**< 0.001**
	Testosterone	-0.000 (-0.000 to 0.000)	0.465	0.000 (-0.000 to 0.000)	0.560
	Fat mass X Testosterone	0.000 (-0.000 to 0.000)	0.826	0.000 (-0.000 to 0.000)	0.783
**Estradiol**	Fat mass	**0.010 (0.007 to 0.013)**	**< 0.001**	.	.
	Estradiol	0.000 (-0.000 to 0.001)	0.234	.	.
	Fat mass X Estradiol	0.000 (-0.000 to 0.000)	0.059	.	.

All models adjusted for age, stature, pubertal status, lean mass, and baseline TBLH BMC.

Total body less head bone mineral content, TBLH BMC; Dehydroepiandrosterone sulphate, DHEAS.

[a] Free Leptin Index log-transformed using the natural log.

Number of children (n) varies from 230 to 380 for different variables: Insulin: n = 380, 194 girls and 186 boys; Free Leptin Index, n = 376, 190 girls and 186 boys; Adiponectin; n = 377, 191 girls and

186 boys; DHEAS: n = 374, 190 girls and 184 boys; Testosterone; n = 231, 113 girls and 118 boys; Estradiol, n = 112, 112 girls and 0 boys.

Table S2.5. Associations between fat mass and endocrine factors (mediator model)

**Exposure**	**Outcome**	**Girls**		**Boys**	
		ß (95% CI)	p-value	ß (95% CI)	p-value
**Fat Mass**	Insulin	**0.260 (0.120 to 0.400)**	**< 0.001**	**0.406 (0.317 to 0.496)**	**< 0.001**
	Free Leptin Index [a]	**0.186 (0.167 to 0.205)**	**< 0.001**	**0.177 (0.162 to 0.193)**	**< 0.001**
	Adiponectin	-0.117 (-0.274 to 0.040)	0.144	-0.102 (-0.263 to 0.060)	0.216
	DHEAS	-0.022 (-0.048 to 0.005)	0.107	-0.001 (-0.031 to 0.029)	0.932
	Testosterone	-5.239 (-12.195 to 1.717)	0.140	**10.310 (0.089 to 20.531)**	**0.048**
	Estradiol	-1.548 (-3.174 to 0.078)	0.062	.	.

All models adjusted for age, stature, pubertal status, lean mass, and baseline TBLH BMC.

Total body less head bone mineral content, TBLH BMC; Dehydroepiandrosterone sulphate, DHEAS.

[a] Free Leptin Index log-transformed using the natural log.

Number of children (n) varies from 230 to 380 for different variables: Insulin: n = 380, 194 girls and 186 boys; Free Leptin Index, n = 376, 190 girls and 186 boys; Adiponectin; n = 377, 191 girls and 186 boys; DHEAS: n = 374, 190 girls and 184 boys; Testosterone; n = 231, 113 girls and 118 boys; Estradiol, n = 112, 112 girls and 0 boys.

Table S2.6. Fixed values used in *Med4Way* analysis

	**Girls**	**Boys**
Fat mass reference value (median value)	7.01	6.48
Fat mass actual value (75th percentile value)	11.40	10.67
Insulin (median value)	5.75	4.88
Log-transformed Free Leptin Index (median value)	2.86	2.53
Adiponectin (median value)	7.87	7.89
DHEAS (median value)	0.69	0.74
Testosterone (median value)	217.52	186.71
Estradiol	6.68	.
Age (median value)	9.73	9.80
Stature (median value)	140.10	141.90
Pubertal status (median value)	Pre-pubertal	Pre-pubertal
Lean mass (median value)	20.48	23.03
Baseline TBLH BMC (median value)	0.64	0.68

Continuous values were mean-centred for entry into analysis.

Dehydroepiandrosterone sulphate, DHEAS; Total body less head bone mineral content, TBLH BMC.

References

1. Bean CG, Pingel R, Hallqvist J, Berg N, Hammarström A. Poor peer relations in adolescence, social support in early adulthood, and depressive symptoms in later adulthood—evaluating mediation and interaction using four-way decomposition analysis. Annals of Epidemiology. 2019;29:52-9.

2. Lee JJ, Valeri L, Kapur K, Ibne Hasan MOS, Quamruzzaman Q, Wright RO, et al. Growth parameters at birth mediate the relationship between prenatal manganese exposure and cognitive test

scores among a cohort of 2- to 3-year-old Bangladeshi children. Int J Epidemiol. 2018;47(4):1169-79.

3. VanderWeele TJ. A unification of mediation and interaction: a 4-way decomposition. Epidemiology. 2014;25(5):749-61.
